# Response: Commentary: Early Risk Detection of Burnout: Development of the Burnout Prevention Questionnaire for Coaches

**DOI:** 10.3389/fpsyg.2020.545159

**Published:** 2020-10-15

**Authors:** Paul Schaffran, Jens Kleinert, Sebastian Altfeld, Christian Zepp, Konrad Wolfgang Kallus, Michael Kellmann

**Affiliations:** ^1^Unit of Sport Psychology, Faculty of Sport Science, Ruhr University Bochum, Bochum, Germany; ^2^Institute of Psychology, German Sport University Cologne, Cologne, Germany; ^3^German Research Centre for Elite Sport Cologne, German Sport University Cologne, Cologne, Germany; ^4^Institute of Psychology, University of Graz, Graz, Austria; ^5^School of Human Movement and Nutrition Sciences, University of Queensland, Brisbane, QLD, Australia

**Keywords:** emotional exhaustion, coaching, stress, recovery, motivation

## Introduction

This commentary refers to the recently published commentary by Lundkvist et al. (2019) in this journal and aims at clearing up an obvious misunderstanding. Lundkvist et al. (2019) critize rightly at the beginning of their commentary that the BPQ-C (Schaffran et al., 2019) “should not currently be used by practitioners and researchers to screen for burnout” (Lundkvist et al., 2019, p. 1). It may have been apparent to the attentive reader that the aim of the questionnaire is not the screening of burnout but much rather the acquisition of critical factors that increase the probability of burnout if combined adversely and persistently. There is no hint to the use of the BPQ-C as screening instrument for burnout in the article (Schaffran et al., 2019). On the contrary, it is suggested that “The BPQ-C should primarily be implemented to detect potential causes of burnout to derive individual preventive measures” (Schaffran et al., 2019, p. 9). Furthermore, in view of the preliminary validation of the internal structure of the questionnaire, it is recommended to use it only in research for the time being.

## Lack of Theoretical Foundation

The first point of criticism that “Schaffran et al.'s definition (and subsequent measurement) of burnout is inadequate” (Lundkvist et al., 2019, p. 1) does not contradict our explanation in the least. It is never stated in the article that the scales are to be understood as a definition of burnout. Instead, our approach aimed at examining and measuring prodromes of burnout which might differ from burnout itself. Further we agree with Lundkvist et al. (2019) that Maslach states the predominantly recognized definition with emotional exhaustion as the key symptom for burnout. Due to this, *Emotional Exhaustion* from the Maslach Burnout Inventory (Maslach et al., 1996) is used as dependent variable in the regression analysis as initial step for the questionnaire development.

The second point of criticism aims at a conflation of “antecedents, outcomes, and the construct of burnout itself” (Lundkvist et al., 2019, p. 1). It is possible that naming the dimensions has led to a misunderstanding. The dimension *Burnout* does not constitute the central characteristic of burnout, but rather show the factors that result from an interaction of constantly high values in the dimension *Pre-Burnout* and low values in the dimension *Resources* (Schaffran et al., 2019). Hence an adverse recovery-stress balance is manifested, which, however, does not represent a distinct indicator for the existence or emergence of burnout, but simply increases the probability of a burnout (Kellmann et al., 2016a). However, even if the dimension *Burnout* does not reflect all central characteristics of burnout, it covers more than simple stress symptoms (e.g., amotivation). The Scale *Fatigue*, as recorded in the BPQ-C, stems from the Recovery-Stress Questionnaire and is relates to “feelings of tiredness and exhaustion, with lack of sleep as well as with overfatigue” (Kallus and Kellmann, 2016, p. 56). *Emotional Exhaustion*, however, is defined as “emotional resources are depleted, workers feel they are no longer able to give of themselves at a psychological level” (Maslach et al., 1996, p. 4). Thus, fatigue encompasses both psychological and physiological components that can develop in direct connection with the coaching activity as well as in other areas of life. Whereas, emotional exhaustion is defined as a psychological symptom and only manifests in the respective work context. In addition, the only moderate correlation (*r* = 0.65) of the two scales does not indicate a large overlap between the two concepts (Schaffran et al., 2019).

The third point of criticism relates to the assumed 3-factor structure of the BPQ-C. The results of the cross-sectional study do not support the assumption that different phases in the development of burnout can be captured with the BPQ-C. However, the relationship between the recovery-stress balance and the development of burnout has already been published in cross-sectional (Altfeld and Kellmann, 2015) as well as in longitudinal studies (Altfeld et al., 2015; Kellmann et al., 2016a).

Regarding the fourth point of criticism, we agree with Lundkvist et al. (2019) that various variables in the burnout research can be causes as well as consequences of burnout. Hence, we see the importance of testing interactions of these constructs with scales that exceed the classical burnout factors. Due to this our approach describes dimensions that are not typically considered in burnout research, which, however, could help to improve the (theoretical) view on burnout. Researching causal relations of different constructs naturally require longitudinal studies, which are still pending in a satisfactory way with the BPQ-C as well as with most other instruments (Raedeke and Kenttä, 2012; Lundkvist et al., 2016).

## Utility as a Screening Tool

The BPQ-C was particularly developed for research interests and is not meant for clinical diagnostics. Moreover, the paper by Schaffran et al. (2019) presents a preliminary version of the BPQ-C which has to be confirmed in replication studies containing further samples. Therefore, the present version of the BPQ-C is not suitable for diagnostic-based interventions.

## Questionnaire Development

Research literature for the development of the questionnaire mainly refers to the development on item level. The approach in the development of the BPQ-C, however, was not the development of a new measurement from scratch, but the combination of concepts or questionnaires with a validated correlation with burnout. The criticism on the rejection of the BPQ-C's one-factor structure in sample 1 is comprehensible from a statistical viewpoint. It does, however, contradict the theoretically grounded, systematic separation between recovery and stress in particular (Kenttä and Hassmén, 1998). Accordingly, the concentration of recovery and stress parameters within a factor was not compatible with the theoretical approach of the *Recovery-Stress Questionnaire* (Jimenez et al., 2016; Kallus, 2016; Kellmann et al., 2016b).

However, it was to be expected that the psychometric properties of the BPQ-C would show weaknesses due to the merging of the contents of different questionnaires and theories. As described above, the BPQ-C should be used for research purposes only at this stage. The correlated residuals in the three-factor structure of the BPQ-C have already been sufficiently argued in the paper (Schaffran et al., 2019) as well as in the manual of the Recovery-Stress Questionnaire (Kallus and Kellmann, 2016). In addition, both the model with and without modifications were presented to allow replication with other data sets.

## Discussion

This commentary shows that area of application and objective of the BPQ-C were misinterpreted. The BPQ-C is no screening instrument that can differentiate context-independently between healthy and ill individuals, even if it is claimed that “studies have provided substantial evidence for the reliability and validity of the BPQ-C as an innovative screening instrument” (Schaffran et al., 2019, p. 9). Rather the BPQ-C focuses on the factor acquisition which can have an impact on coaches in particular and influence the emergence and development of burnout in coaches. This is also emphasized within the scope of Schaffran et al. (2019, p. 9) article: “The BPQ-C may emerge as a valuable tool in understanding influencing factors for burnout in coaches.” While already validated methods like the Maslach Burnout Inventory (Maslach et al., 1996) or the Shirom Melamed Burnout Questionnaire (Kushnir and Melamed, 1992) show more stable psychometric characteristics, they cannot capture the specific parameters of the coach context. The inclusion of these factors, however, is essential for a successful prevention of burnout since burnout per definition is strongly context-dependent (World Health Organization, 2018) and hence influencing factors in various occupations can differentiate immensely.

## Author Contributions

PS is the main author who initiated the commentary, wrote the first draft, and has been editing all other contributors' drafts and comments. MK, JK, CZ, KK, and SA were involved in the design of the paper and have been commenting and editing the paper throughout the whole process.

## Conflict of Interest

The authors declare that the research was conducted in the absence of any commercial or financial relationships that could be construed as a potential conflict of interest.
